# Data for drugs available through low-cost prescription drug programs are available through pharmacy benefit manager and claims data

**DOI:** 10.1186/1472-6904-12-12

**Published:** 2012-06-22

**Authors:** Vivienne J Zhu, Anne Belsito, Wanzhu Tu, J Marc Overhage

**Affiliations:** 1 Regenstrief Institute, Inc, Indianapolis, Indiana, USA; 2 Indiana University School of Medicine, Indianapolis, IN, USA; 3 Siemens Healthcare, Malvern, PA, USA

**Keywords:** Low-cost prescription program, Oral antihyperglycemic agents, Pharmacy benefit manager, Claims data

## Abstract

**Background:**

Observational data are increasingly being used for pharmacoepidemiological, health services and clinical effectiveness research. Since pharmacies first introduced low-cost prescription programs (LCPP), researchers have worried that data about the medications provided through these programs might not be available in observational data derived from administrative sources, such as payer claims or pharmacy benefit management (PBM) company transactions.

**Method:**

We used data from the Indiana Network for Patient Care to estimate the proportion of patients with type 2 diabetes to whom an oral hypoglycemic agent was dispensed. Based on these estimates, we compared the proportions of patients who received medications from chains that do and do not offer an LCPP, the proportion trend over time based on claims data from a single payer, and to proportions estimated from the Medical Expenditure Panel Survey (MEPS).

**Results:**

We found that the proportion of patients with type 2 diabetes who received oral hypoglycemic medications did not vary based on whether the chain that dispensed the drug offered an LCPP or over time. Additionally, the rates were comparable to those estimated from MEPS.

**Conclusion:**

Researchers can be reassured that data for medications available through LCPPs continue to be available through administrative data sources.

## Background

When pharmacies dispense a medication for a patient who has a drug benefit, they typically submit an electronic transaction to a pharmacy benefit management (PBM) adjudication system as a method to confirm eligibility and to request payment. The PBM returns a transaction which contains status data about the adjudication and later transfers the transaction data to the payer who contracted with them for services.

In 2006, pharmacies introduced low-cost prescription programs (LCPP) offering selected generic medications that included those for common diseases, such as diabetes, hypertension, and asthma, for $5 or less for a 30-day supply (they sometimes offer a 90-day supply for $10 to $15) [1]. Researchers and others who rely on claims data became concerned that, since the dispensing pharmacy would be unlikely to receive additional reimbursement from the payer and there may be direct and indirect costs associated with submitting the claim, the pharmacy might often not submit a claim when the patient purchased one of these low cost prescriptions using cash. [2,3] Failing to submit claims for these drugs, many of which are commonly used, would diminish the value of administrative data sources for research [4]. Even if the drug in question was not the primary focus of a study, important confounding or comorbidity data could be lost.

In order to determine whether LCPPs have an effect on the availability of low-cost medication dispensing data through claims, we analyzed data from a large health information exchange and compared the proportion of patients receiving each oral hypoglycemic medication available through LCPPs at pharmacy chains with and without LCPPs. We also compared the proportion of patients who had at least one prescription for an oral hypoglycemic medication before and after pharmacies implemented LCPPs. Our hypothesis was that, if patients pay cash for medications available through LCPPs, the proportion of patients appearing to use these medications would appear lower for chains with LCPP compared to chains without these programs. Similarly, we would expect the proportion of patients receiving each of these medications to appear to fall after the pharmacy chains implemented LCPPs.

## Methods

This study was approved by the Institutional Review Board of Indiana University. We chose to base our evaluation on patients with type 2 diabetes, a common disease that requires treatment with medications chronically. We chose to study patients with a specific condition in order to allow us to estimate usage rates (proportion of patients who received a prescription for a drug) which we could compare across chains with and without LCPPs from 2008 to 2010, before and after the LCPP implementation from 2002 to 2010, as well as with estimates based on the most recent Medical Expenditure Panel Survey, a nationally representative survey of medical care use and expenditures.

### Indiana network for patient care

The Indiana Network for Patient Care (INPC) is an operational regional health information exchange, which collects and transfers healthcare information electronically across organizations within a region, community or hospital system [5]. The INPC services more than 75 participating hospitals as well as laboratories, radiology centers, public health departments, long-term care facilities, payers, some pharmacies, and PBMs, and the INPC has served Indianapolis for more than 15 years [1,6]. In particular the INPC includes pharmacy claims data from the largest public and private payors as well as medication history data from PBMs obtained *via* the Surescripts network. Surescripts is the country’s largest electronic prescribing network providing electronic access to prescription information. It connects all of the nation’s major chain pharmacies, many of the nation’s leading payers, and over 10,000 independent pharmacies nationwide. The medication data usually include patient identifying data, such as name, gender, ethnicity, and address; drug data, including a coded identifier, whether the drug dispensed was branded, and the number of days’ supply dispensed; and dispensing pharmacy information, including the National Council for Prescription Drug Programs (NCPDP) pharmacy code. We map the coded drug identifiers (almost always National Drug Codes but sometimes pharmacy specific codes) indirectly to the RxNORM codes (a standardized nomenclature for clinical drugs and drug delivery devices developed and maintained in the National Library of Medicine), which allowed us to aggregate drugs at the level of active ingredients. We selected commonly used antidiabetic medications (First DataBank Standard Therapeutic Class Code: 71), including selected oral hypoglycemic agents (OHA) therapeutic classes: sulfonylurea, biguanides, thiazolidinediones, α-glucosidase inhibitors, meglitinides, dipeptidyl-peptidase-4 inhibitors, and antidiabetic combinations.

### Pharmacy data

Using the NCPCP pharmacy database, we aggregated dispensing locations into chains by store name. We defined an LCPP as a program that offered 30-day supplies of medications for $5 or less and which did not have obvious barriers to participation, such as annual membership fees or difficult enrollment processes. In addition to reviewing the grey and published literature [4,7], we reviewed both the current and past versions of the websites for each of the 14 chains in our database to determine which offered an LCPP and which antidiabetic medications were currently included in the program. [7,8] Using these data, we identified five major chains which implement LCPP and provide generic antidiabetic medication (glimepiride, glipizide, glyburide,and metformin). While the chains implemented their LCPPs at slightly different times, they essentially started in the 4th quarter of 2006, and most were implemented by the 4th quarter of 2007.

### Measurements

In order to construct a measure of the rate of medication use for each category, we identified all patients in the Indianapolis Metropolitan Statistical Area (MSA) who had at least one clinical encounter with type 2 diabetes (ICD-9-CM codes 250.X0 or 250.*X*2 as the primary diagnosis) from 2008 to 2010. We then assigned each patient who had at least 3 medication dispensing records (assuming that patients with a chronic disease on chronic medications would have a minimum of 4 dispensing events over the course of 1 year, even if they received 90-day supplies and were only being treated with 1 drug) to the pharmacy chain through which they received their prescriptions. We excluded patients who received prescriptions from more than 1 chain during the study period from the cohort in order to eliminate any cross-over effects. Next, we computed the rate at which each drug was used in each pharmacy chain cohort of patients. If a patient had at least 1 dispensing event for the drug, we included them in the numerator, while the total number of diabetic patients attributed to the pharmacy chain was used as the denominator. Using the same approach, we measured the proportion of patients using individual OHA longitudinally (2002 to 2010) for a single payer.

In order to obtain comparable independent estimates of rates of use of these drugs nationally, we extracted data from the Medical Expenditure Panel Survey (MEPS) 2008 Prescribed Medicines dataset (file: HC-118A). [9] The MEPS study is a large-scale survey of families and individuals, their medical providers, and employers across the United States by the Agency for Healthcare Research and Quality (http://www.meps.ahrq.gov/mepsweb/). The estimated proportion of diabetes patients using each OHA drug in the year 2008 was used as a reference measure. We selected patients who had at least 1 diagnosis code for diabetes and used the MEPS weightings to project the proportion of diabetic patients receiving each active ingredient to the U.S. population.

### Analysis

Primarily, we compared the rates at which a specific medication was dispensed to diabetic patients between chains offering and not offering an LCPP. In the generalized linear mixed models (GLMM), the dispensing event of low-cost medications was the dependent variable, and the low-cost program was used as the independent variable. Odds ratios (OR) were used to quantify the magnitude of associations between these 2 variables. Both adjusted and unadjusted associations were estimated. Patient age, gender, race, and pharmacy chain were used as covariates (fixed effects) for the adjusted analysis. Patients were included in the model as the random effect.

In order to take account into secular trends, we secondarily evaluated longitudinal trends of the proportion of patients using low-cost medication in major chains with and without LCPP. The interrupted time series analyses with control group were performed to assess the immediate changes of proportion of low-cost OHA use after LCPP implementation and to analyze if any detectable change was caused by the LCPP. For this analysis, the proportion of low-cost OHA use was measured quarterly. Changes of proportion of low-cost OHA use and changes of the slopes between pre-intervention period (2002, quarter 1 to 2006, quarter 4) and post-intervention period (2007, quarter 1 to 2010, quarter 4) were estimated and compared for chains with LCPP (intervention) and without LCPP (control).

## Results

**Figure 1:**
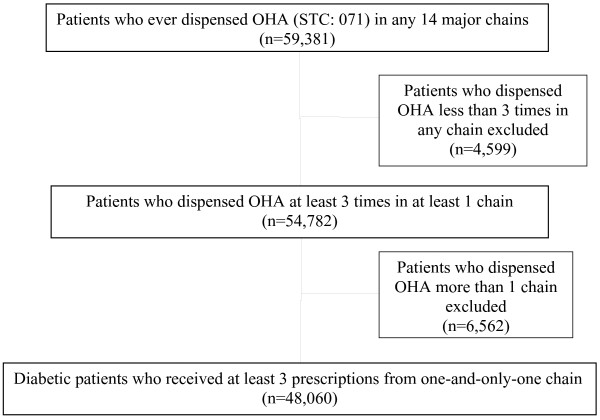
Patient selection.

**Figure 2:**
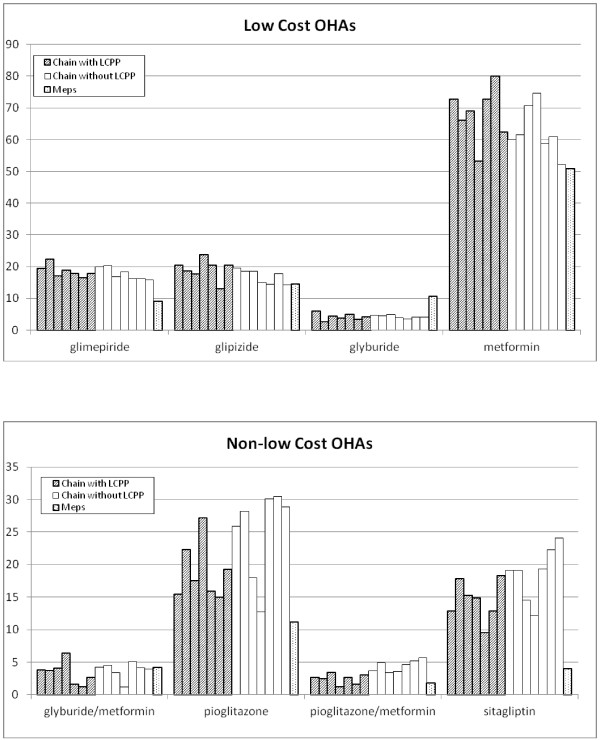
Proportion of patients using selected oral hypoglycemic agents(OHA) by chain 2008–2010.

We identified 48,060 diabetic patients who received at least 3 prescriptions from one-and-only-one of 14 pharmacy chains (4,129 individual pharmacies) which cover more than 95% of INPC patients who had OHA dispensing records (Figure 1). The demographic characteristics of the patient cohorts were similar across chains (Table 1). Among a total of 620,648 OHA dispensing events, 268,473 were dispensed from chains without an LCPP and 352,175 were dispensed from chains with an LCPP. Overall, the percentage of dispensing events of low-cost medications was higher in pharmacy chains which have low-cost programs: 71.7% *vs.* 66.1%. After controlling for patient gender, race, and chain, chains with an LCPP are more likely to dispense an OHA which is available as a low-cost medication (OR: 1.21,95% CI [1.19,1.22], p < 0.0001). Proportions of patients for which each OHA was dispensed are similar for drugs across chains whether they offer an LCPP or not (Figure 2). In addition, the proportions of diabetic patients receiving each medication based on projections from the MEPS data were similar to those in our Indianapolis cohorts (Table 2).

**Table 1:** Patient characteristics

**Chains**	**Number of Patients**	**Age of dispensing**		**Female**	**White**
		**(Mean**±**SD)**			
**Chain1**†	424	63.9	± 12.0	50.70%	83.20%
**Chain2**	489	61.2	± 13.0	43.90%	87.50%
**Chain3**	693	66.4	± 10.4	51.50%	81.70%
**Chain4**†	727	60.7	± 13.1	45.20%	91.20%
**Chain5**†	810	53.5	± 12.3	52.30%	84.20%
**Chain6**‡	966	57.3	± 12.6	45.60%	83.60%
**Chain7**	1,708	60.8	± 11.0	40.10%	89.60%
**Chain8**	2,892	60.8	± 10.2	40.80%	91.80%
**Chain9**	3,461	64.0	± 11.0	43.10%	87.80%
**Chain10**†	3,932	59.1	± 12.8	51.00%	87.70%
**Chain11**†	5,623	57.6	± 12.3	50.80%	82.40%
**Chain12**	7,971	58.2	± 12.8	51.70%	75.10%
**Chain13**	9,882	66.6	± 10.6	43.10%	85.10%
**Chain14**†	16,788	59.1	± 12.9	49.00%	79.00%
**MEPS**	13.7million	60.7	± 12.8	44.2%	66.8%

† Chain with a LCPP.

‡ Chain with LCPP for Chlorpropamide only.

**Table 2:** Proportion of patients using selected oral hypoglycemic agent (OHA) by chain

**Ingredient**	**MEPS**	**Chain14**	**Chain11**	**Chain1**	**Chain10**	**Chain4**	**Chain2**	**Chain5**	**Chain13**
Chlorpropamide *	0.2	0.04	0.01	0	0.02	0	0	0	0.03
Glimepiride *	9.1	17.1	19.4	18.8	18.0	17.8	22.4	16.5	19.9
Glipizide *	14.5	17.6	20.3	23.8	20.4	20.3	18.6	12.9	19.6
Glyburide *	10.7	4.3	5.9	3.7	4.9	4.2	2.6	3.4	4.6
Metformin *	51.0	69.1	72.8	53.3	72.8	62.4	66.2	80.0	59.9
Acarbose	0.1	0.4	0.3	0.9	0.4	0.2	0.2	0.2	0.4
Glimepiride/Pioglitazone	0.3	0.2	0.26	1.1	0.2	0.1	0.8	0.1	0.2
Glimepiride/Rosiglitazone	0.5	0.4	0.17	0.4	0.4	0.6	0.4	0.2	0.5
Glipizide/Metformin	0.1	0.6	0.56	0.9	0.5	0.8	0.6	0.2	0.7
Glyburide/Metformin	4.1	4.0	3.8	6.3	1.5	2.6	3.6	1.2	4.1
Pioglitazone	11.1	17.5	15.4	27.1	15.9	19.2	22.2	14.9	25.8
Pioglitazone/Metformin	1.8	3.3	2.5	1.1	2.6	3.0	2.4	1.6	3.6
Repaglinde	0.8	0.8	0.5	0.9	0.6	0.9	0.8	0.1	1.0
Rosiglitazone	0.5	3.0	2.3	6.8	3.1	3.0	3.4	2.3	5.6
Rosiglitazone/Metformin	0.6	1.4	1.1	2.3	1.6	1.5	1.8	1.2	2.0
Sitagliptin	4.0	10.8	9.0	12.2	9.4	12.7	12.2	8.8	14.3
Sitagliptin/Metformin	1.1	4.9	4.3	2.8	4.5	6.7	6.1	4.5	5.3
Low-cost OHA		85.3	89.0	78.5	89.0	83.2	83.4	90.0	79.0
Non-low-cost OHA		55.3	33.8	52.1	34.4	43.4	43.7	29.3	52.2

*low-cost oral antidiabetic medication.

**Figure 3:**
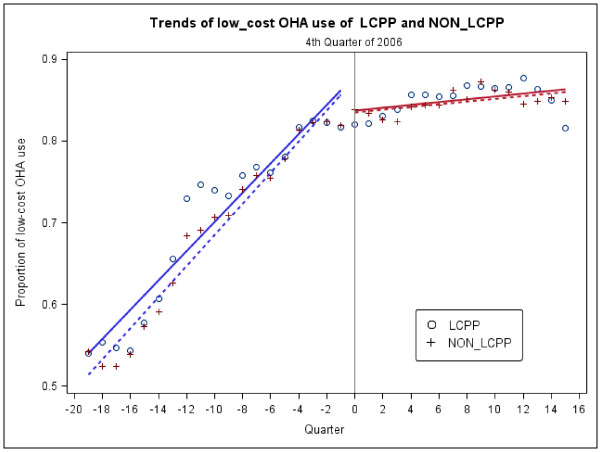
Trend of proportion of patients using low-cost oral hypoglycemic agent (OHA) in chains with or without a low-cost program from 2002 to 2010.

From the longitudinal (2002–2010) dataset, a total of 18,775 patients were identified (14,220 for LCPP with 154,525 low-cost OHA dispensing events, and 4,555 for non-LCPP with 74,738 low-cost OHA dispensing events). Figure 3 demonstrates the results of segmentation regression for 36-quarter intervals. In the LCPP group post-LCPP implementation and controlling for baseline trends, no sudden changes of the proportion of low-cost medication use were found (p = 0.14), and a slight decline of slope was observed (−0.01, p < 0.0001). Similarly, in the non-LCPP group, there was also no significant drop of low-cost medication use (p = 0.06); however, there was a change in slope (−0.01, p < 0.0001) over time. In the segmentation regression with two groups, there was no difference between the groups in prior trend (p = 0.35). After LCPP implementation, the immediate change was not significantly different for the LCPP and non-LCPP groups (p = 0.88), and there was no significant difference between the LCPP and non-LCPP groups in the change in slope (p = 0.57).

## Discussion

To our knowledge, this is the first study that investigated whether LCPPs decrease data availability of low-cost medication dispensing events. We did not find any evidence to support the concern voiced by some researchers that pharmacies may not be sending claims for low-cost generic medications to PBMs. The estimated proportion of patients receiving each anti-diabetic medication was comparable not only across chains, regardless of whether they offered an LCPP, but also before and after the time frame in which chains implemented LCPPs. In addition, from the longitudinal data, we further observed no significant difference in changes in the level and slope of the proportion of low-cost medication use after LCPP implementation between chains with and without LCPP.

In the longitudinal data set, low-cost OHA use has been rapidly increasing from 2002 to 2006. A slower growing of the proportion of low-cost medication use has been observed in chains with and without LCPP in the beginning of 2007. Possible explanation could be an amount of patients dispensed preferred branded antidiabetic medications even the insurance program has encouraged using generic OHAs. In addition, some patients in this population might be eligible for Medicare Part D program when it was implemented in 2006, which we might not capture complete medication dispensing information for these patients.

The proportions for anti-diabetic drugs which are not included in the LCPPs were similar between chains offering LCPPs and those that do not, which increases our confidence in our estimation process. In addition, the similarities of the proportion of patients using each OHA between our estimates based on pharmacy claims and estimates from the MEPS data increase support our belief that our claims-based estimates are reasonable.

From these experiences, we did not find evidence to support the concern that the PBMs do not capture dispensing events of low-cost medications in chains with LCPP. We speculate that because of the level of automation of the submission process in most pharmacies and the minimal cost of transmitting these transactions that, unless a patient pays cash and specifically requests that the pharmacy not share the data with the patient’s payor, the pharmacy will send the claim to the patient’s PBM. To not send in the claim, the technician or the pharmacist would have to change the patient’s payer to "cash", which would require more work than submitting a claim to the third-party when the patient has active prescription drug coverage in their profile.

### Limitation

It is possible that the central Indiana population may not be representative of other populations but the demographics are similar to the overall US population. We only studied OHAs in diabetic patients who have active insurance coverage. Findings may not apply to other medications in the LCPP or to patients without active insurance coverage. In addition, we were not able to directly measure the proportion of patients receiving OHAs to compare with the estimate based on claims transactions, but we believe that our approach of comparing the proportions for patients receiving medications from chains with and without LCPPs are a good proxy. Note that provider orders would not provide a good gold standard since so many prescriptions go unfilled. We might not have identified all diabetic patients and may have included some Type 1 diabetics, but this should not introduce any systematic bias. Further, assignment of patients to pharmacy chains was reasonable based on at least 3 OHA dispensing events during the study period, which might exclude information from patients who had less than three OHA dispensing records in the INPC. However, this assignment may provide information for a more stable study population for each chain.

## Conclusion

Our findings should reassure researchers that dispensing data for medication available through LCPPs are not selectively excluded from PBM or claims datasets.

## Competing interest

The authors declare that they have no competing interests.

## Authors’ contributions

VZ participated in literature research, study design, data analysis/interpretation, and manuscript preparation. AB participated in study design and data acquisition. WZ participated in study design and data analysis. JMO led manuscript definition of intellectual content, study design, data analysis/interpretation, and manuscript preparation. All authors read and approved the final manuscript.

## Pre-publication history

The pre-publication history for this paper can be accessed here:

http://www.biomedcentral.com/1472-6904/12/12/prepub

## Ackowledgements

We would like to thank Roberta Ambuehl (senior data analyst) at the Regenstrief Institute, Inc.
